# Adsorption of Lead Ions by a Green AC/HKUST-1 Nanocomposite

**DOI:** 10.3390/nano10091647

**Published:** 2020-08-21

**Authors:** Paria Soleimani Abhari, Faranak Manteghi, Zari Tehrani

**Affiliations:** 1 Research Laboratory of Inorganic Chemistry and Environment, Department of Chemistry, Iran University of Science and Technology, Narmak, Tehran 1684613114, Iran; paria96soleimani@gmail.com; 2 College of Engineering, Centre for NanoHealth, Institute of Life Science 2, Swansea University, Singleton Park, Swansea SA2 8PP, UK

**Keywords:** metal organic framework, active carbon, heavy metal, low-cost adsorbents, lead sensor, *Cortaderia selloana*

## Abstract

A new nanocomposite consisting of activated carbon (AC) from the *Cortaderia selloana* flower and copper-based metal-organic framework (HKUST-1) was synthesized through a single-step solvothermal method and applied for the removal of lead ions from aqueous solution through adsorption. The nanocomposite, AC/HKUST-1, was characterized by Scanning Electron Microscopy (SEM), X-ray Diffraction (XRD), Fourier Transform Infrared (FTIR), and Energy-Dispersive X-ray Spectroscopy (EDX) methods. The SEM images of both HKUST-1 and AC/HKUST-1 contain octahedral crystals. Different factors affecting adsorption processes, such as solution pH, contact time, adsorbent dose, and initial metal pollution concentration, were studied. The adsorption isotherm was evaluated with Freundlich and Langmuir models, and the latter was fitted with the experimental data on adsorption of lead ion. The adsorption capacity was 249.4 mg g^−1^ for 15 min at pH 6.1, which is an excellent result rivalling previously reported lead adsorbents considering the conditions. These nanocomposites show considerable potential for use as a functional material in the ink formulation of lead sensors.

## 1. Introduction

The different polluting agents released from human industrial and agricultural activities are placing the environment under immense strain. Amongst the various water pollution sources, heavy metals are considered the most dangerous contributors. Common water pollutant heavy metals such as copper, bismuth, lead, mercury, cadmium, and nickel have densities over 5 g cm^−3^. Due to their stability and biodegradability, they can cause severe environmental problems and endanger human health [1,2]. Lead is one of the most significant contributors to heavy metal contamination and is problematic in part because it is ubiquitous, reported to occur in water, air, vegetables, and soil [3]. As such, the maximum level of lead pollutant in drinking water permissible by the WHO and the Environmental Protection Agency (EPA) are 50 and 15 µg/L, respectively [4]. Lead is one of the most toxic elements and its devastating effects on human health are well documented; studies showed that lead can attack the brain and nervous system, cause intellectual disabilities (IDs) and behavioral disorders, and damage multiple organ systems including the kidneys and liver. The symptoms of lead poisoning include vertigo, insomnia, anemia, headaches, mortality, weakness, and hallucinations [4,5,6]. The situation is compounded further by lead being widely used across various industries such as printing, photographic materials, explosive production, ceramic and glass manufacturing, metal plating, and finishing [6,7]. Considering lead’s toxic nature and its abundance, it is imperative to accurately detect its presence and concentration to mitigate its adverse impacts. As such, the development and advancement of effective and accurate lead sensors is important and will have countless applications. 

Looking at the various treatment systems available to remove heavy metals from aqueous solutions such as ion exchange, reverse osmosis, electrocoagulation, precipitation, membrane filtration, and electrochemical methods, adsorption remains superior due it is simplicity, effectiveness, low cost, and environmentally friendly contribution [8,9,10,11]. Several adsorbents such as zeolite [12], activated carbon (AC) [13], and silica gel [14] are used for the removal of heavy metals from an aqueous solution that are not as efficient as metal organic frameworks (MOFs) due to their low adsorption capacities and surface area [15]. Recently, researchers studied nanomaterials and found that they exhibit superior properties and show potential for increasing the adsorption of heavy metals in contrast to traditional adsorbents [16].

MOFs are some of the best materials used for adsorption. New MOFs have a high surface area, large pore volume, network design, large surface-to-volume ratio, and stable porosity [17,18]. MOFs are also applied widely in different fields, i.e., sensing [19], drug delivery, storage [20,21], catalysis [22,23], gas storage, separation [24], liquid and gas adsorption, and many more. MOFs are crystalline porous materials that consist of secondary building units, such as metal ions, and organic linkers connected into a three-dimensional network [17].

Plant biomass exists widely in nature and can be used as a readily available and low-cost adsorbent. These adsorbents have an unrivalled microstructure that is particularly favorable in applications such as catalysis and adsorption [25], and their chemical combination consists of cellulose and lignin with various polar functional groups such as carboxylic, ether, hydroxyl, carbonyl and phenolic groups [25,26,27]. *Cortaderia selloana* is a tussock and perennial grass native to South America; however, it is found in many countries and areas around the world. This plant can endure a vast variety of environmental conditions [28]. There are a few reports on the application of *Cortaderia selloana* flower spikes in the field of absorbance for removing heavy metals. To develop the MOF applications, a range of materials such as multi-walled carbon nanotubes (MWCNT), active carbon (AC), biomaterials, graphene oxide (GO), and nanofibrous membranes can be used as the supporting bed to grow improved MOF nanocrystals [29,30]. For instance, Mahmoodi et al. studied the AC/MIL-101 composite as a bio-based novel green adsorbent [31], Jia et al. synthesized low-cost absorption materials based on *Cortaderia selloana* flower spike biomass for dye removal [25], and Wang et al. successfully synthesized graphene/copper benzene-1,3,5-tricarboxylate metal organic framework (HKUST-1) in a direct one-step reaction [32]. There is a paucity in literature regarding the characterization and application of AC/copper-based metal-organic framework (HKUST-1) nanocomposites for heavy metal adsorption.

Improved sensing abilities of sensor devices are in part dictated by the ability of the functional material to uptake target molecules. Therefore, materials that exhibit notable adsorption properties can be exploited to improve the sensing abilities of sensors. As such, we aimed to produce and characterize a green nanocomposite from plant biomass as a potential functional material in the development of lead sensor technology. An AC/copper-based MOF was synthesized using the solvothermal method, then characterized, and employed as an adsorbent to remove lead ions (Pb (II)) from simulated solutions. The effects of variables such as the contact time, pH, and adsorbent dosage were investigated. Equilibrium adsorption isotherm and kinetic models were also studied to evaluate the experimental data.

## 2. Materials and Methods

### 2.1. Materials

Copper nitrate trihydrate, Cu(NO_3_)_2_·3H_2_O; lead nitrate, Pb(NO_3_)_2_; benzene-1,3,5-tricarboxylic acid, C_6_H_3_–1,3,5-(COOH)_3_, (H_3_BTC) 98%; and methanol, CH_3_OH 99.5% were all purchased from Merck and Sigma-Aldrich (Darmstadt, Germany).

### 2.2. Characterization Techniques

The powder X-ray diffraction analysis was carried out using a Phillips X-pert diffractometer (PXRD) (Almelo, The Netherlands) with monochromatic Cu-Kα radiation (λ = 1.54056Å). The morphology of surfaces was studied using a JEOL 630 -FSEM (Tokyo, Japan). The synthesized materials were controlled using a Nicolet 100 FTIR (Chicago, IL, USA) in the range of 4000–400 cm^−1^ by the KBr pellet method [33]. The inductively-coupled plasma optical emission spectrometry (ICP-OES) on a Varian Vista-PRO apparatus (Pau, France), equipped with a charge-coupled detector, was used to determine the concentration of heavy metal ions. Energy-Dispersive X-ray Spectroscopy (EDX) spectroscopy data were processed with a SAMx and P_X10p software (France). AC/HKUST-1 was analyzed by a UV–Vis spectrophotometer (Shimadzu UV-1700) (Kyoto, Japan). The sonication processes in this study were conducted using a Misonix Sonicator 2200 power output (maximum 300 W at 50/60 kHz) (New York, NY, USA).

### 2.3. Methods

#### 2.3.1. Preparation of Active Carbon from *Cortaderia selloana*

*Cortaderia selloana* flowers were obtained from the garden of Iran University of Science and Technology’s campus and were sun-dried for five successive days to entirely dehydrate. The dried sample was then pulverized using a ball mill (Retsch MM 400) (Haan, Germany). The ground flowers (6 g) were soaked in 50 mL of 50% *w*/*v* phosphoric acid solution at 30 °C for 48 h. After filtration, the raw material was then carbonized in a muffle furnace at 300 °C for 2 h in an argon atmosphere. After cooling, the carbonized material was washed with 200 mL of hot distilled water, and then dried for 2 h at 120 °C. The dried activated carbon was weighed to determine the percentage yield [34]. The calculation and result are given in Equation (S1), Supplementary Materials.

#### 2.3.2. Preparation of AC/HKUST-1

The nanocomposite AC/HKUST-1 was prepared by a one-step solvothermal method. In summary, 0.264 g (1.25 mmol) of H_3_BTC was dissolved in 7.5 mL of ethanol and was mixed with 0.545 g (2.25 mmol) of Cu(NO_3_)_2_·3H_2_O and then dissolved in 7.5 mL deionized (D.I.) water [32]. The mixture was ultrasonicated for 20 min to obtain a homogenous solution, then 4 mg of AC was added and shaken. The homogeneous solution was transferred to a Teflon-lined stainless-steel reactor and heated to 120 °C for 24 h. After cooling, the obtained precipitate was carefully collected by centrifugation, washed with D.I. water and ethanol several times, and dried at 80 °C for 10 h in a vacuum.

#### 2.3.3. Lead Adsorption Experiments

To investigate the adsorption process and the removal of Pb (II) ions from aqueous solution by AC/HKUST-1, adsorption experiments were carried out by adding 10 mg of the adsorbent to 50 mL lead ion solution obtained from Pb(NO_3_)_2._ The solution containing controlled pH was centrifuged at high speed for 10 min. The amount of lead adsorbed by AC/HKUST-1 and the Pb (II) removal efficiency were calculated using Equations (1) and (2), respectively.

(1) $$q_{t}=\frac{\left(C_{0}-C_{t}\right)V}{M}$$

(2) $$\;\;\;R=\frac{\left(C_{0}-C_{e}\right)}{C_{0}}\times 100$$

where *q_t_* (mg g^−1^) is the amount of absorbate per amount of absorbent at time *t*, *R* is efficiency, and *C_e_* (mg L^−1^) and *C*_0_ (mg L^−1^) are the equilibrium and initial concentrations of Pb (II) ions, respectively. The solution volume is shown by *V* (L), and *M* (g) demonstrates the mass of the absorbent.

## 3. Results and Discussion

### 3.1. Characterization of Adsorbent and Its Components

Adsorption performance of AC/HKUST-1 in an aqueous solution of Pb (II) was studied by considering some parameters including contact time with lead ions in solution, effect of pH, initial concentration of the solution, and adsorbent dose determined at room temperature. We found that AC/HKUST-1 demonstrates admissible adsorption capacity for lead ions compared to the recently reported adsorbents. HKUST-1, as one of the oldest reported MOFs, was prepared with a simple synthetic process [35] and characterized by methods described in this section.

**Figure 1:**
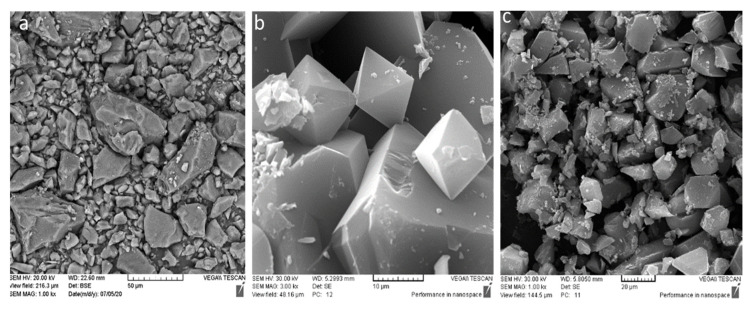
The SEM images of (**a**) Active Carbon (AC), (**b**) Copper benzene-1,3,5-tricarboxylate metal organic framework (HKUST-1), and (**c**) AC/HKUST-1.

The SEM images show the surface structural characteristics of the AC (Figure 1a), HKUST-1 (Figure 1b), and AC/HKUST-1 (Figure 1c), and how visual changes can be observed at the SEM-level in the composition; the pseudo-octahedral crystals of HKUST-1 are shown in Figure 1b, which are also observed through the AC particles in Figure 1c. The pore structure of AC is detailed in Supplementary Materials Table S1.

**Figure 2:**
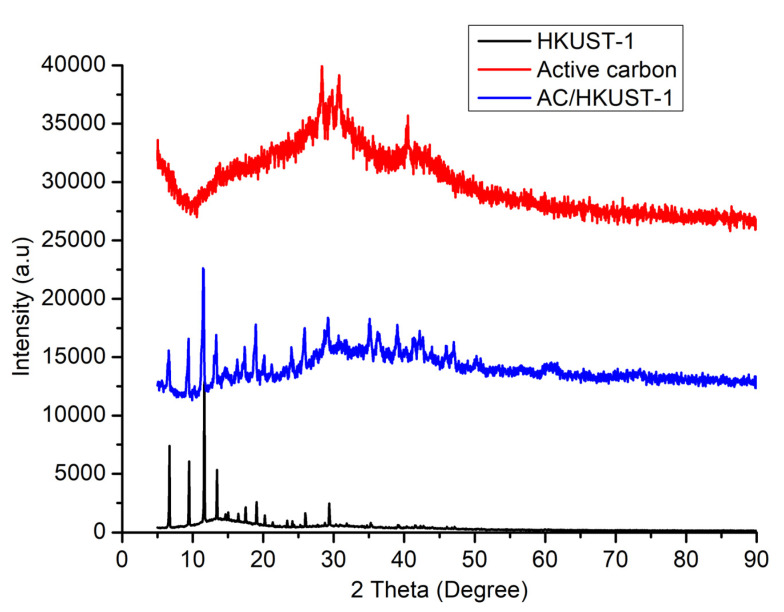
XRD patterns of AC (top), AC/HKUST-1 (middle), and HKUST-1 (bottom).

The XRD pattern of HKUST-1 compared with the simulated pattern is illustrated in Supplementary Materials Figure S1, and the patterns of nanocomposite and its components are illustrated in Figure 2. The two figures confirm the presence of HKUST-1 and AC in the composite.

### 3.2. Factors Affecting Adsorption Processes

#### 3.2.1. Effect of pH on Pb (II) Adsorption

**Figure 3:**
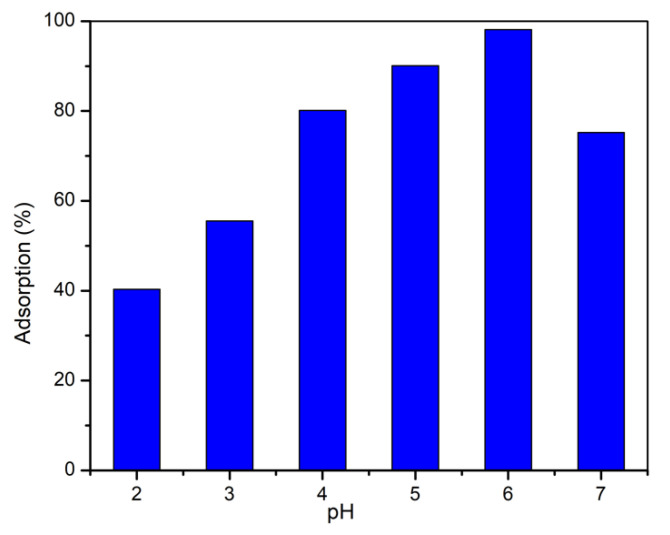
Effect of the solution pH on Pb (II) adsorption on AC/HKUST-1 nanocomposite.

One of the most critical factors that can improve the capacity of lead adsorption is the solution pH. The effect of pH on the adsorption of Pb (II) on AC/HKUST-1 is shown in Figure 3. First, 50 mL of 50 ppm lead solution and 10 mg adsorbent were mixed to check the adsorption efficiency of AC/HKUST-1. We found that when the pH is lower than three, the removal efficiency is very low. Hydronium ion (H_3_O^+^) and metal ions are located in the surface adsorption sites. However, at pH ≥ 7, precipitation occurred, and through further increases in pH and reduction of H_3_O^+^, an electrostatic interaction occurred between Pb (II) ions and the functional sites of AC/HKUST-1. Therefore, at pH 6.1, we identified the highest rate of lead ions adsorption by AC/HKUST-1.

#### 3.2.2. Effect of Contact Time (Adsorption Kinetics)

**Figure 4:**
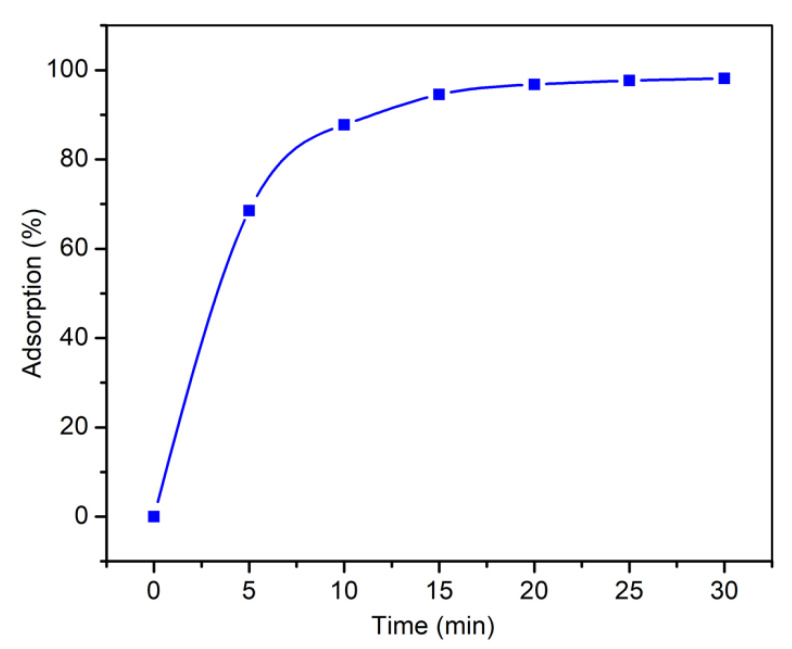
Effect of the contact time on Pb (II) adsorption on AC/HKUST-1.

Adsorption kinetics studies reveal key information about the mechanisms behind an adsorption system. Hence, the removal of lead ions by nanocomposite AC/HKUST-1 was checked as a function of contact time. The adsorbent (10 mg) was added to a 50 mL lead ion solution at pH = 6 and *C*_0_ = 50 ppm, then placed in an ultrasonic bath at 25 °C for a certain time. As shown in Figure 4, the adsorbent adsorbed lead ions in the first 15 min, after which an equilibrium was reached. This may potentially be due to an abundance of available adsorption sites and lead ions occupying most of the active sites [18].

To obtain more comprehensive data on the adsorption kinetics to further interpret the adsorption process, kinetic methods including pseudo-first order and pseudo-second-order models were investigated by the adsorption data of Pb (II) by Equations (3) and (4).

(3) $$log\left(q_{e}-q_{t}\right)=logq_{e}-\left(\frac{k_{1}}{2.303}\right)t$$

(4) $$\frac{t}{q_{t}}=\frac{1}{k_{2}q_{e}^{2}}+\left(\frac{1}{q_{e}}\right)t$$

where *q_e_* and *q_t_* are the amounts of lead adsorbed (mg g^−1^) at equilibrium and at time *t* (min), respectively; *k*_1_ (min^−1^) and *k*_2_ (g mg min^−1^) are the constants for pseudo-first-order and pseudo-second-order reactions, respectively. The results of the kinetic models are shown in Table 1.

**Figure 5:**
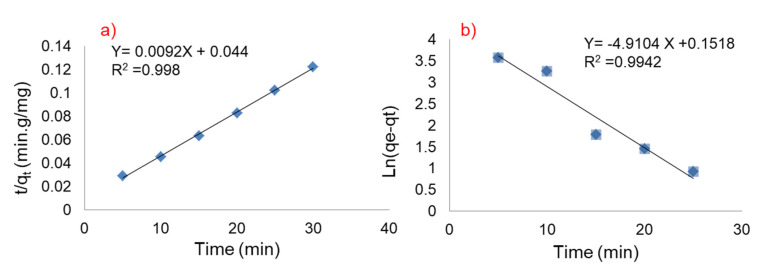
(**a**) Pseudo-second order and (**b**) pseudo-first-order kinetics models for adsorption of Pb (II) on AC/HKUST-1 at different initial concentrations.

As shown in Figure 5, the value of correlation coefficient, *R*^2^, for the pseudo-second-order model and pseudo-first-order are 0.998 and 0.994, respectively. Therefore, the calculated value of pseudo-second-order equilibrium adsorption is higher than that of the pseudo-first-order model for lead ions. In addition, the *R*^2^ for the pseudo-second-order kinetic model demonstrates that the adsorption method is initiated by chemical reactions between the water pollutant Pb (II) ions and the active adsorbent sites [33].

#### 3.2.3. Effect of Lead Concentration

**Figure 6:**
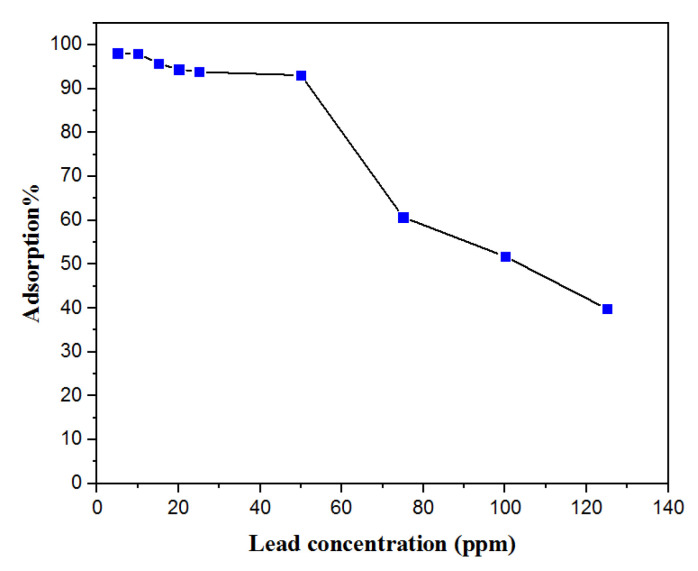
Effect of primary concentration of Pb (II) adsorption onto AC/HKUST-1 at 25 °C.

To evaluate the performance of the adsorbent, different concentrations (5, 10, 15, 20, 25, 50, 75, 100, and 125 ppm) of aqueous Pb (II) were prepared and the adsorption percentage was measured. Ten mg of adsorbent was added to every solution and kept in an ultrasonic bath for 30 min at room temperature to obtain a homogenous solution. Observable in Figure 6, the results showed that with increasing concentration of Pb (II) from 5 to 50 ppm, the adsorption decreased very slightly from ~95% to ~90%, whereas a more significant decrease in adsorption was observed in the range of 50–70 ppm. Therefore, we concluded that the adsorbent is more efficient for aqueous lead solutions at concentrations of 5–50 ppm.

#### 3.2.4. Effect of Adsorbent Dose

**Figure 7:**
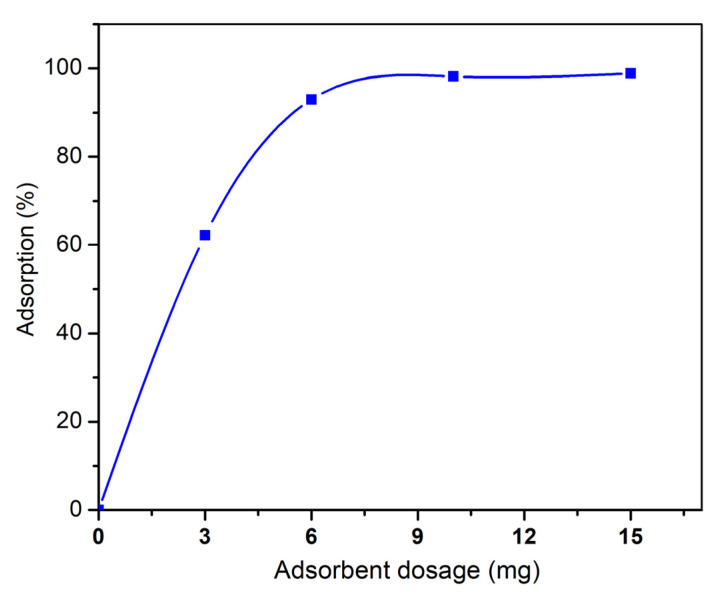
Effect of adsorbent dosage on the adsorption percentage of Pb (II) onto AC/HKUST-1.

The efficacy of AC/HKUST-1 dose on adsorption was investigated at pH 6 and room temperature; the results are shown in Figure 7. We showed that with increasing quantity of adsorbent, the Pb (II) removal efficiency increased. This behavior could be attributed to the availability of sufficient active sites during the Pb (II) adsorption process that remained unsaturated. At an adsorbent dosage of 7.4 mg, an adsorption of 97% was achieved.

#### 3.2.5. Comparison between Nanocomposite and Its Components

To measure the capacity of Pb (II) adsorption by HKUST-1 and AC, 10 mg of AC and HKUST-1 were added separately to 50 mL aqueous solution at pH = 6 and *C*_0_ = 50 ppm, then placed in an ultrasonic bath at 25 °C for 30 min. The adsorption capacities of the AC/HKUST-1, HKUST-1, and AC are shown in Table 2.

The results highlighted that the adsorption capacity of the nanocomposite (AC/HKUST-1) is significantly higher than that of its components, HKUST-1 and AC.

**Scheme 1:**
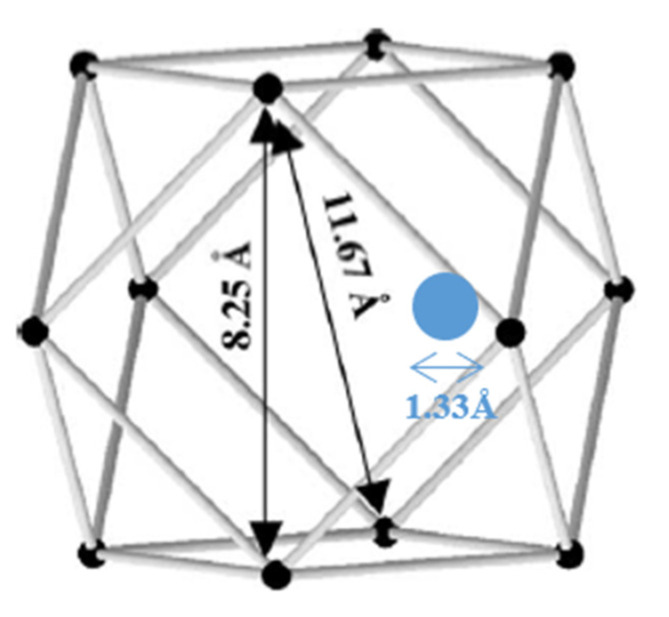
The structure and dimension of HKUST-1 pores shown in black and Pb (II) in blue.

Scheme 1 illustrates the polyhedron structure and dimensions [36] of HKUST-1 and the Pb (II) fitting into the HKUST-1 pores.

### 3.3. Adsorption Isotherm

**Figure 8:**
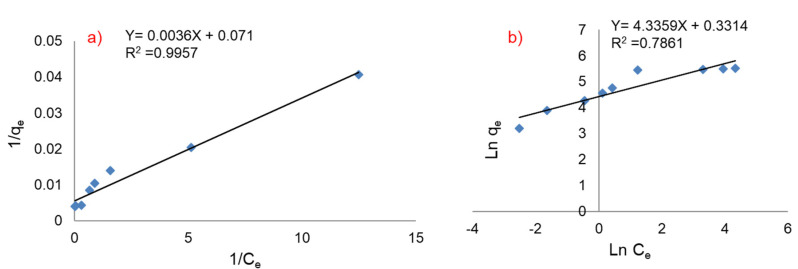
Two-parameter isotherm models for adsorption of Pb (II) on AC/HKUST-1: (**a**) Langmuir and (**b**) Freundlich.

At several initial concentrations of AC/HKUST-1, the amount of lead adsorption was measured, and the results are shown in Figure 8. Adsorption isotherms can demonstrate the action and reaction between adsorbent and adsorbate, and are considerable on different adsorbents [37]. It was measured using two-parameter isotherm models Freundlich and Langmuir and the experimental data were analyzed using these models. Both were evaluated for adsorption isotherm following Equations (5) and (6).

(5) qe=kFCe1n

where *C_e_* (mg L^−1^) is the equilibrium concentration of Pb (II) in aqueous solution, and *n* and *k_F_* (L mg^−1^) are Freundlich constant and adsorption capacity, respectively.

(6) $$\frac{C_{e}}{q_{e}}=\frac{C_{e}}{q_{m}}+\frac{1}{K_{L}q_{m}}$$

where *q_e_* (mg g^−1^) is the amount of Pb (II) adsorbed at equilibrium, *q_m_* (mg g^−1^) is the maximum amount of adsorption capacity, *C_e_* (mg L^−1^) is the equilibrium concentration of Pb (II) in water solution, and *K_L_* is an equilibrium constant of Langmuir model. The results of two isotherm models are shown in Table 3.

The *R*^2^ for the Langmuir and Freundlich models are 0.99 and 0.79, respectively, as illustrated in Figure 8.

Therefore, since the calculated *R*^2^ for the Langmuir adsorption isotherm is higher than that for the Freundlich lead ions, the adsorption likely corresponds to the Langmuir model.

**Figure 9:**
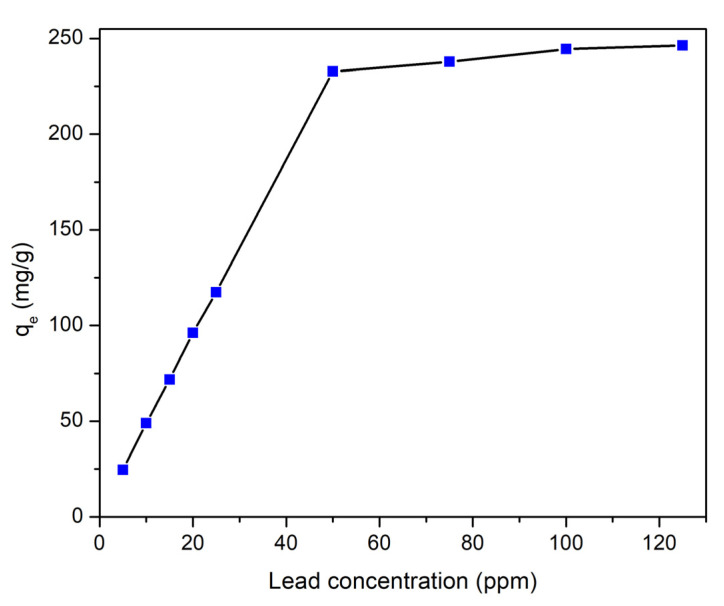
Effect of adsorbent dosage on Pb (II) ions adsorption capacity.

Figure 9 illustrates the effect of adsorbent amount on the adsorption capacity of AC/HKUST-1. Accordingly, the highest adsorption capacity of Pb (II) is 249.4 mg g^−1^.

### 3.4. A Comparative Adsorption Study with Various Ions

**Figure 10:**
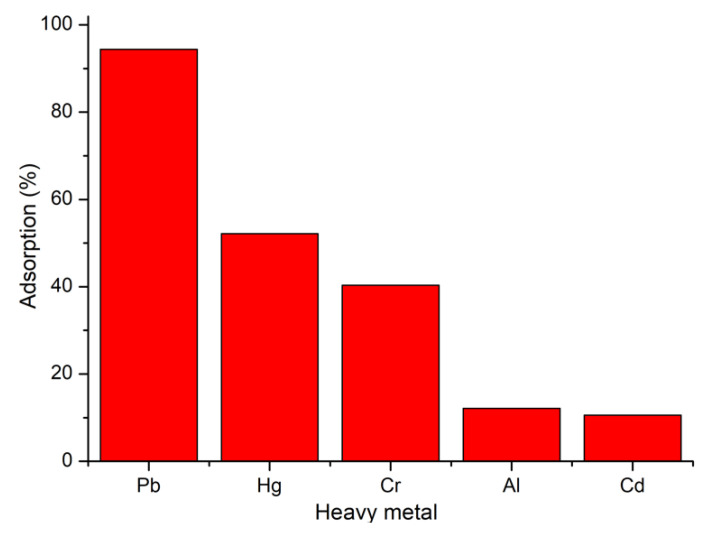
Comparative adsorption of various toxic ions.

The efficiency of the adsorbent, AC/HKUST-1, was investigated for various metal ions. In comparison, several metal ions comprising Pb^2+^, Hg^2+^, Cr^3+^, Al^3+^, and Cd^2+^ in the solution were prepared, and their results illustrated in Figure 10 confirm the largest absorption of Pb (II).

### 3.5. Reusability Potential

**Figure 11:**
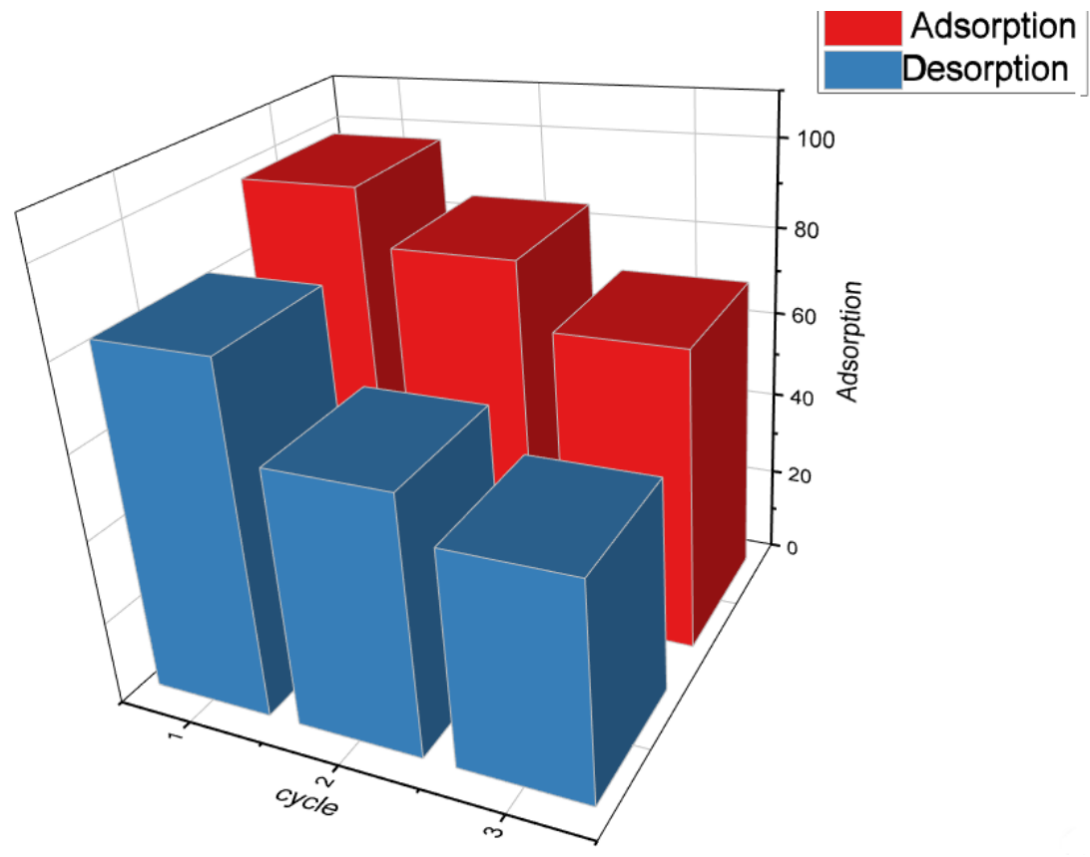
Cycles of adsorption-desorption on AC/HKUST-1.

One of the characteristics of the adsorbent is its reusability in the sorption procedure. To determine this function of the adsorbent, AC/HKUST-1, the lead metal ions were applied in three steps of the adsorption and desorption cycle. Desorption was performed by adding 2 mL of deionized water to 10 mg of adsorbent, and the resulting solution was placed into an ultrasonic bath and dispersed for 20 min. The released Pb (II) ions were then measured using Inductively Coupled Plasma (ICP) Spectroscopy. The results are shown in Figure 11 and after three cycles, the adsorption of AC/HKUST-1 is less than the first cycle.

### 3.6. Adsorption Mechanism

To study the performance and adsorption mechanism of AC/HKUST-1, FTIR spectroscopy was applied; the results are shown in Figure S2. All absorption bands of active carbon and HKUST-1 exist, which showed that the coordination position of HKUST-1 and active carbon is protected in the composite [38].

In addition, energy-dispersive X-ray spectroscopy (EDX) analysis was conducted to investigate the flow of Pb (II) adsorption, shown in Figure S3a–c, in which the elemental analysis of the nanocomposite after adsorption, nanocomposite before adsorption, and AC are compared. The presence of lead ion in the adsorbent structure is observed after adsorption.

To complete the investigation, the AC/HKUST-1 nanocomposite was synthesized, characterized, and applied to remove harmful Pb (II) ions from an aqueous solution. Any associated factors that could affect this process were investigated.

The nanocomposite exhibited a maximum adsorption capacity of 249.4 mg g^−1^ at optimum conditions of pH 6.1, which is an excellent result rivalling those of TMU-5 (Zn(oba)(4-bpdh)0.5]n·(DMF)y) [39] (Table 4). Whereas TMU-5 showed a slightly higher adsorption capacity of 251 mg g^−1^, the optimum conditions necessary to achieve this consisted of a pH 10, with reports that decreasing pH resulted in a decrease in the adsorption capacity of the adsorbent. Additionally, the materials used in TMU-5 are not commercially available. However, our nanocomposite works at a much lower pH of 6.1, near neutral, and the MOFs used are commercially available. These results make nanocomposite an attractive material to use, especially as a functional material in lead sensors.

## 4. Conclusions

In this research, we identified a method for successfully producing the nanocomposite AC/HKUST-1 to adsorb lead ions from an aqueous solution. The novelty of this work lies in the straightforward and eco-friendly preparation of the nanocomposite AC/HKUST-1 based on commercially available materials such as HKUST-1 and active carbon from *Cortaderia selloana* flowers. Evaluation of diverse parameters, such as dosage, pH, initial concentration of lead ions, and time of lead adsorption, confirmed a number of attractive properties of AC/HKUST-1, namely its adsorption capacity; the nanocomposite was able to adsorb 97% of lead ions from an aqueous solution with the maximum adsorption capacity of Pb (II) onto AC/HKUST-1 being 249.4 mg g^−1^. Additionally, tests conducted with the adsorption of other toxic metal ions confirmed that the nanocomposite produced is relatively selective for lead ions. Combining this with its proven reusability, AC/HKUST-1 has the potential to serve as a valuable functional material in lead sensors.

Future work will focus on harnessing the properties and adsorption capacity of this material and integrating it into sensor systems through ink formulations as a functional material. Successful integration will mean that the resulting sensor has the potential to exhibit enhanced sensitivity, attributed to AC/HKUST-1’s adsorption capacity of lead ions. Producing this through screen-printing will enable the up-scaling for potential commercial applications and so the resulting sensor systems can be produced at low cost and high volume, serving as an important development in lead sensor technology.

The application of sensitive lead sensors manufactured in volume at low cost has great commercial interest due to an array of potential applications such as industrial processing, biotechnology and medical diagnostics, particularly in the development of point of care (POC) devices.

## Figures and Tables

**Table 1:** Parameters and kinetics models for the adsorption of Pb (II) by AC/HKUST-1.

Kinetics Model	*k*	*R* ^2^	*q_e_*
Pseudo-first order	0.34	0.994	135.69
Pseudo-second order	1.09 × 10^−2^	0.998	250

**Table 2:** Comparison of the adsorption capacities of HKUST-1, AC, and AC/HKUST-1.

	HKUST-1	AC	AC/HKUST-1
**Adsorption (%)**	72.98	40.58	97

**Table 3:** Constants of Langmuir and Freundlich models for adsorption of Pb (II) ions.

	Langmuir Model			Freundlich Model		
**Adsorbent**	***q_m_* (mg/g)**	***k*_1_ (L/mg)**	***R*^2^**	***k_F_***	***n***	***R*^2^**
**AC/HKUST-1**	227.77	0.507	0.99	76.39	3.017	0.79

**Table 4:** A comparison between reported adsorbents for Pb (II) ion.

Adsorbents	Adsorption Capacity of Pb (II) Ions (mg g^−1^)	Time of Adsorption (min)	Optimum pH	Ref
**TMU-5 (Zn(oba)(4-bpdh)0.5]n·(DMF)y)**	251	15	10	[39]
**HKUST-1 TMW@H_3_PW_12_O_40_**	98	10	7	[40]
**UiO-66NHC(S)NHMe**	232	240	-	[41]
**AMOF-1**	71	1440	-	[42]
**Cu-terephthalate Metal Organic Framework (MOF)**	80	120	7	[43]
**Dy(BTC)(H_2_O)(DMF)_1.1_**	5	10	6.5	[44]
**ZnO/AC from coconut shell**	76.66	-	5.6	[45]
**AC from rice straw**	36.05	-	5	[46]
**AC from palm shell**	95.20	-	3.0 and 5.0	[47]
**AC/HKUST-1**	**249.4**	**15**	**6.1**	**This work**

## Supplementary Materials

The following are available online at https://www.mdpi.com/2079-4991/10/9/1647/s1, Figure S1: XRD of as-synthesized and simulated HKUST-1; Figure S2: FTIR spectrum of HKUST-1 and AC/HKUST-1; Figure S3: EDX spectra of AC, AC/HKUST-1, AC/HKUST-1 before and after adsorption; Table S1: Brunauer–Emmett–Teller (BET) Analysis, and total pore volume of AC.
