# Supplementary material for: Adsorption of Lead Ions by a Green AC/HKUST-1 Nanocomposite
Source: Nanomaterials (Basel). 2020 Aug 21;10(9):1647. doi: 10.3390/nano10091647 (PMC7559321; doi:10.3390/nano10091647)
Supplement: Supplementary file 1 [file nanomaterials-10-01647-s001.pdf]

nanomaterials

Adsorption of Lead Ions by a Green AC/HKUST-1 Nanocomposite

Paria Soleimani Abhari [1], Faranak Manteghi [1,*] and Zari Tehrani [2,*]

[1] Research Laboratory of Inorganic Chemistry and Environment, Department of Chemistry, Iran University of Science and Technology, Narmak, Tehran, 1684613114, Iran; paria96soleimani@gmail.com
[2] College of Engineering, Centre for NanoHealth, Institute of Life Science 2, Swansea University, Singleton Park, Swansea, UK, SA2 8PP
* Correspondence: f_manteghi@iust.ac.ir (F.M.) and Z.Tehrani@swansea.ac.uk (Z.T.)

The equation (SI1) represents how percentage yield is calculated

$$Percentage\ yield\ (\%) = \frac{yield(\%)}{mass\ of\ raw\ material\ (g)} \times 100 \qquad (E1)$$

The XRD pattern of HKUST-1 compared with the simulated pattern is illustrated in SI Figure S1

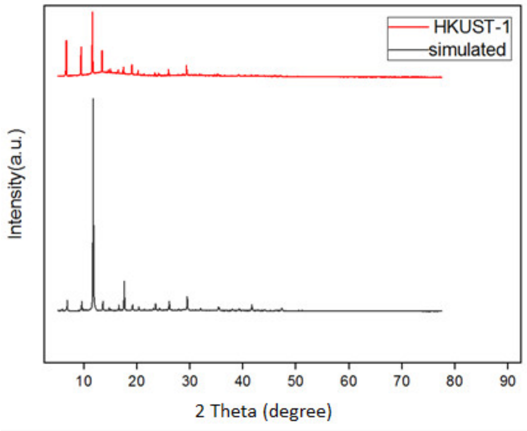

Figure S1. XRD of as-synthesized (red, top) and simulated (black, bottom) HKUST-1.

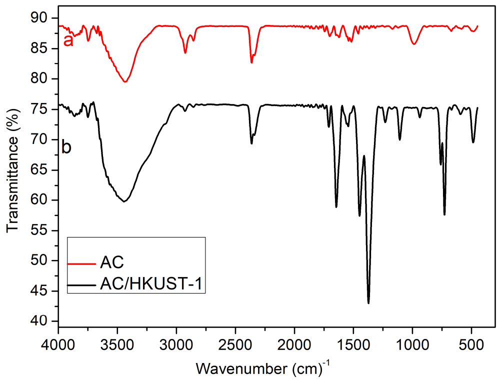

Figure S2. FTIR spectrum of (a) HKUST-1 and (b) AC/HKUST-1.

To study the performance and adsorption mechanism of AC/HKUST-1, the FTIR spectroscopy was applied, which is shown in Figure S2. The broad peak of 3500–3000 cm^{-1} is ascribed to the stretching vibration of water molecules. For AC/HKUST-1 composite, all absorption bands of active carbon and HKUST-1 exist, which shows that the coordination position of HKUST-1and active

carbon is protected in the composite. The absorption peak of AC, which is in the range of 3420–3510 cm^{-1} is characteristic of OH group stretching vibration from water or carboxyls adsorbed on the activated carbons, which is also seen in Figure S2-a. The absorption bands at 3000–2930 cm^{-1} in the IR spectrum is for C-H stretching. A band matches to the characteristic absorption of C=C around 1630 cm^{-1}. Finally, the peak at 500 cm^{-1} is related to metal-oxygen coordination bond of HKUST-1.

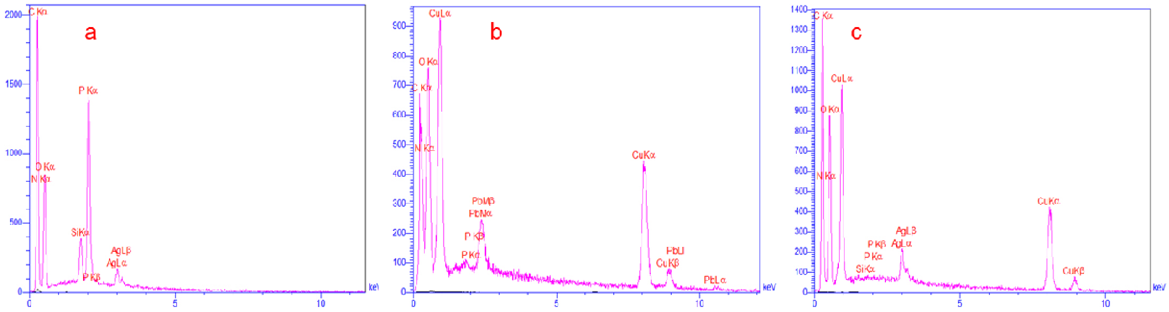

Figure S3. EDX spectra of (a) AC , (b) AC/HKUST-1 after adsorption ,(c) AC/HKUST-1 before adsorption,.

Energy-dispersive X-ray spectroscopy (EDX) analysis was carried out to investigate the flow of Pb(II) adsorption, and the results are shown in Figure S3 (a-c), in which the elemental analysis of the nanocomposite after adsorption (a), nanocomposite before adsorption (b), and AC (c) is observed. The peaks of lead ion clearly shown in Figure S3 (a) demonstrate the presence of lead ion in the adsorbent structure.

The pore structure and specific surface area of AC are presented in Table S1.

Table S1. BET Analysis, and total pore volume of AC.

	S_{BET} (m^2/g)	V_m (cm^3/g)	*Total pore dimension* (nm)
AC	145.76	33.488	3.206
